# Extrinsic apoptosis participates to tail regression during the metamorphosis of the chordate *Ciona*

**DOI:** 10.1038/s41598-023-48411-y

**Published:** 2024-03-08

**Authors:** Gabriel Krasovec, Cécile Renaud, Éric Quéinnec, Yasunori Sasakura, Jean-Philippe Chambon

**Affiliations:** 1 Institut de Systématique, Evolution, Biodiversité, Sorbonne Université, Muséum National d’Histoire Naturelle, CNRS, EPHE, F-75252 Paris Cedex 05, France; 2 Shimoda Marine Research Center, University of Tsukuba, Shizuoka, Japan; 3 CRBM, Université de Montpellier, CNRS, 34000 Montpellier, France; 4 Present Address: Université Paris Cité, CNRS, Institut Jacques Monod, F-75013 Paris, France

**Keywords:** Cell biology, Developmental biology, Molecular biology

## Abstract

Apoptosis is a regulated cell death ubiquitous in animals defined by morphological features depending on caspases. Two regulation pathways are described, currently named the intrinsic and the extrinsic apoptosis. While intrinsic apoptosis is well studied and considered ancestral among metazoans, extrinsic apoptosis is poorly studied outside mammals. Here, we address extrinsic apoptosis in the urochordates *Ciona*, belonging to the sister group of vertebrates. During metamorphosis, *Ciona* larvae undergo a tail regression depending on tissue contraction, migration and apoptosis. Apoptosis begin at the tail tip and propagates towards the trunk as a polarized wave. We identified *Ci-caspase 8/10* by phylogenetic analysis as homolog to vertebrate caspases 8 and 10 that are the specific initiator of extrinsic apoptosis. We detected *Ci-caspase 8/10* expression in *Ciona* larvae, especially at the tail tip. We showed that chemical inhibition of *Ci-caspase 8/10* leads to a delay of tail regression, and *Ci-caspase 8/10* loss of function induced an incomplete tail regression. The specificity between apoptotic pathways and initiator caspase suggests that extrinsic apoptosis regulates cell death during the tail regression. Our study presents rare in vivo work on extrinsic apoptosis outside mammals, and contribute to the discussion on its evolutionary history in animals.

## Introduction

Apoptosis is a regulated cell death allowing the elimination of unwanted cells during various morphogenetic process such as embryogenesis, metamorphosis or homeostasis^1^ and able to influence its immediate cellular environment^2^. Apoptosis is characterised by a conserved set of morphological features (loss of adhesion, cells shrinking, chromatin condensation, membrane blebbing, fragmentation in apoptotic bodies) depending, at the molecular level, on the caspases multigene family^3–5^. Based on these morphological and molecular features, apoptosis has been detected in most metazoan phyla^6–10^. Currently, two main apoptotic regulation pathways are described, acknowledged as the intrinsic (formerly known as mitochondrial pathway) and the extrinsic apoptosis (previously named death receptors pathway) by the most recent Nomenclature Committee on Cell Death (NCCD-2018)^11^. Each of these apoptotic signalling pathways presents the particularity to depend on specific initiator caspases having long pro-domains CARD (Caspase Recruitment Domain) or DED (Death Effector Domain) for the intrinsic and extrinsic apoptotic, respectively. Initiator caspases operate upstream of the regulation and next activate the common downstream executioner caspases.

The characterisation of intrinsic apoptosis in nematode, fly and vertebrates makes it the best known among animals^3,12,13^. Even if recent research suggests divergences between intrinsic apoptosis^14^, this pathway share the specificity to be initiated by a caspase with a pro-domain CARD activated by an APAF-1 protein and is considered ancestral and conserved among metazoans^11,15^.

In mammals, extrinsic apoptosis is initiated by perturbations of the extracellular microenvironment that trigger the binding of specific ligands like CD95 to the TNFr (Tumor Necrosis Factor receptors)^11,16^. The activated TNFr recruits FADD (FAS-Associated Death Domain) or TRADD (Tumor necrosis factor Receptor type 1-Associated Death Domain) which bind to the pro-caspases 8 or 10, respectively. The resulting proteolytic platform called DISC (Death-Inducing Signalling Complex) allows next the fully activation of the initiator caspases.

In opposite to intrinsic apoptosis, extrinsic apoptosis is not present neither in fly nor in nematode and most knowledge about extrinsic apoptosis come from mammals. Consequently, despite the in-depth understanding of extrinsic apoptosis in mammals, this pathway remain largely unexplored at a large evolutionary scale, excepted few punctual works made on cephalochordates, echinoderms or cnidarians^17,18^. Even if a previous study on Amphioxus suggest presence of extrinsic apoptosis in chordates, current knowledge is insufficient to understand the operation and evolution of extrinsic apoptosis among metazoans. However, in the last decades, genomic and transcriptomic analysis identified numerous of proteins potentially involved in apoptosis regulation, including DED-caspases, TNFr, TRADD or FADD like proteins suggesting the presence of extrinsic apoptosis in several animal groups, including ascidians^17,19–21^.

Ascidians, as the sister group of vertebrates, offers an ideal opportunity to analyse the evolution of molecular pathways in chordates, including vertebrates. The ascidian *Ciona* presents a bi-phasic life cycle; sessile adults release gametes in environment where fertilization occurs, followed by a rapid embryogenesis that give rise to a swimming tadpole larva. After a short swimming period, the larvae settle to a substrate and undergo a metamorphosis. The most dramatic event of this phenomenon is the tail regression, depending on multiples factors and molecular pathways including, among others, actomyosin dependant contraction of epidermal cells, GABA, ERK, or D-serine^22–27^. Importantly, tail regression is characterised by a polarised apoptotic wave starting at the tip of the tail and propagates toward the trunk^7^. However, the apoptotic pathway triggering cell death has not been investigated yet while a previous study identified several potential apoptotic actors in *Ciona* based on sequence similarity with mammals, including a caspase with DED domain^28^.

In this study, we took advantage of the easily predictable apoptotic profile during larvae metamorphosis to explore the initiation of apoptosis by focusing on the unique DED-caspase found in the *Ciona* genome, *Ci-caspase 8/10* (gene model KH.C8.550)^28,29^. We first clarified the evolutionary relationship between *Ciona* and vertebrates’ caspases using phylogenetic analysis. We characterised expression of *Ci-caspase 8/10* in larvae, and discovered that apoptotic wave propagation and tail regression is delayed in larvae exposed to a caspase 8 inhibitor. In addition, loss of function of *Ci-caspase 8/10* using Crispr/CAS9 technology significantly increased the number of larvae presenting an incomplete tail regression phenotype. Globally, we demonstrated here that Ci-caspase 8/10 participates to apoptosis regulation during *Ciona* tail regression, arguing in favour of extrinsic apoptosis implication in the process. In addition, comparative effects of various specific caspase inhibitors indicated that among initiator caspases of *Ciona*, Ci-caspase 8/10 has a central role in apoptosis regulation. Ultimately, our data suggest a conservation of the extrinsic apoptosis between ascidians and vertebrates, and also at the chordates scale according to previous work conducted on *Amphioxus*.

## Materiel and methods

### Animals

Wild type *Ciona intestinalis* Type A from Onagawa-bay (Miyagi, Japan) and Onahama-bay (Fukushima, Japan) were cultivated by the National BioResource Project (Japan) and then maintained in seawater at the Shimoda Marine Reasearch Station (Tsukuba University Shimoda, Japan). Wild type *Ciona intestinalis* Type B collected on the field by the Biological Sample Collection Service of the Biological Station of Roscoff (Sorbonne University, Roscoff, France) were maintained in artificial seawater at the Aquatic Model Animal Facility and Engineering of the Institute of Biology Paris-Seine (Sorbonne University, Paris, France). In laboratory, *Ciona* were maintained at 18 °C in 35‰ salinity artificial seawater in a constant light condition to prevent spawning. Eggs and sperm were surgically collected from gonadal ducts, followed by cross-fertilisations in plastic Petri dishes.

Caspases inhibitors treatments and corresponding TUNEL labelling, in situ hybridization of Ci-caspase-8/10 like and TNF receptors, and real-time PCR were conducted on *Ciona* type B. Loss of function experiment and corresponding TUNEL labelling were made on *Ciona* type A. Genome used for phylogenetic analysis and primers design is the genome of *Ciona* type A^29^. 

### Pharmacological treatments

Swimming larvae were distributed in plastic Petri dishes post-hatching. When about 70% of the larvae had settled (eye assessment), the supernatant was replaced by filtered ASW containing specific chemicals or DMSO as control. Chemicals were stored in DMSO at − 20 °C and used at 10 μM of final concentration. Chemicals used were the pan-caspases inhibitor Z-VAD-fmk (Sigma-Aldrich; Merck KGaA), the caspase-2 inhibitor Z-VDVAD-fmk (Sigma-Aldrich; Merck KGaA), the caspase-8 inhibitor Z-IETD-fmk (Sigma-Aldrich; Merck KGaA), the caspase-9 inhibitor Z-LEHD-fmk (Sigma-Aldrich; Merck KGaA), and the caspase-10 inhibitor Z-AEVD-fmk (Sigma-Aldrich; Merck KGaA).

### TUNEL labelling

TUNEL labelling were done using the In Situ Cell Death Detection Kit (Roche) as previously described^30^, or with the Click-iT^®^ TUNEL Alexa Fluor^®^ Imaging Assay (Invitrogen) according to manufacturer instructions.

### Real-time PCR

RNA was extracted using the RNAqueous™-Micro Total RNA Isolation Kit (ThermoFisher Scientific), followed by a DNAse treatment made with the TURBO DNA-*free*™ Kit (ThermoFisher Scientific) and finally purified a second time using the RNeasy MinElute Cleanup Kit (Qiagen). All steps were done according to the manufacturer's protocol.

Real-time PCR was performed using the SYBR Green Supermix (Biorad) with a Biorad’s thermal cycler using the following profile: 95 °C for 10 min; 40 cycles of amplification with successively 95 °C for 15 s, 60 °C for 10 s, and 72 °C for 20 s; one cycle for melting curve analysis with an acquisition every 0.5 °C from 65 °C to 95 °C to verify the presence of a single product. Each assay included a no-template control for each primer pair, and five successive dilutions to determine the Ct values and the reaction efficiencies. Real-time PCR reactions were done in triplicate. Gene expression level was normalized using *Ci-actin* as reference gene as previously described^31–33^. Primer pair used for Ci-caspase 8/10 are: forward 5’-AAGACTGCTTTGTGTGCGTG-3’, reverse 5’-GGCAGGCTTGGAAGAAAAATAT-3’. PCR products length were between 140 and 160 bp.

### Whole in situ hybridization (WISH)

We performed WISH as previously described^30^. Concerning settled larvae used for *Ci-caspase 8/1*0 WISH, we manually removed the tail tunic with forceps as we already done^30^, allowing the complete elimination of the well-known strong background generated by this tissue. TNF receptors WISH were conducted on dechorionated larvae.

### Statistic and phylogenetic analysis

Statistical differences were evaluated by Wilcoxon Mann Whitney test using the software R 2.14.1. Effects were considered significant with a p-value < 0.05 and statistical significance is notated in figures by an *.

Multiple alignments of protein sequences were generated using the MAFFT 7^34^ and Gblocks 0.91b^35^ was used to removed vacancies and blur sites. Phylogenetic analyses were carried out by Maximum-Likelihood (ML) method under LG model using PhyML 3.1^36^, combined ML tree search with 1000 bootstrap replicates. Bayesian analyses were performed using MrBayes 3.2.6^37^ under mixed model. One fourth of the topologies were discarded as burn-in values, while the remaining ones were used to calculate the posterior probability. The run was carried out for 500 000 generations with 5 randomly started simultaneous Markov chains (1 cold chain, 14 heated chains) and sampled every 100 generations.

### Microscopy and images acquisition

Images of Figs. 2 and 3 were taken with a BX61 (Olympus) and images of Fig. 4 an AxioImager Z1 (Carl Zeiss MicroImaging) microscopes. Images from Supplementary Fig. 2 were taken with a DM5000B (Leica) microscope. Images from Supplementary Fig. 3 were taken with a Leica SP5 confocal microscope. Images were analysed with ImageJ 1.46a-1 and AxioVision Rel.4.6 softwares. Samples were mounted in Mowiol 4–88 (Calbiochem) or CitiFluor AF1 (Science Services).

### Microinjection and loss of functions

Eggs were dechorionated and injected with a mixture composed of 250 ng/µL of crRNA, 1 ng/µL of tracrRNA, 10 µg/µL of Cas9 IDT, and blue dye^38^. Larvae used for experiment are the F0 developed from eggs successfully injected with the mix (sorted thank to the presence of the dye). DNA were extracted from about 50 larvae labelled by the dye next day after injection. The crRNA number 1 was 5’-TTTTTCTTCCAAGCCTGCCA-3’ and genomic region of Ci-casp.8/10 targeted by the gRNA was amplified by PCR (forward 5’-ctgcaggaattcgatGTGTATGGTGTTGATGGCTTGT-3’; reverse 5’-atcgataagcttgatAACATCATCAACTGGAGCGGAA-3’) using the ExTaq (Takara-bio). The crRNA number 2 was 5’- TCGCTTTCAACATCATCAAC -3’ and genomic region of Ci-casp.8/10 targeted by the gRNA was amplified by PCR (forward 5’-ctgcaggaattcgatTTCAGACCAGGCATGTGC-3’; reverse 5’-atcgataagcttgatAAGAAGTCAGACTCACTT-3’). PCR products were run on SDS poly-acrylamide gel electrophoresis to verify size heterogeneity induced by mutations. Then, PCR products were subcloned into the *Eco*RV site of pBluescript SKII ( +) and sequenced by the conventional Sanger method to evaluate the mutation rate.

#### In silico* docking experiment*

Caspases amino acids sequences were converted to .pdb format using the Expasy webserver https://swissmodel.expasy.org/interactive. SMILES formats of inhibitor chemicals were obtained on PubChem database and converted to .pdb format with the NovoPro Labs Smiles2PDB webserver https ://www.novoprolabs.com/tools/smiles2pdb. In silico analysis were run with iGemdock^39^ under Drug screening settings for 70 generations and population size of 200.

### Ethics declarations

According to the French legislation (Décret n° 2013–118 du 1er février 2013 relatif à la protection des animaux utilisés à des fins scientifiques), experiments on *Ciona intestinalis* do not require an approval from Animal Ethics Commission because its not a “live vertebrate animals” and not a “live cephalopods”.

## Results

### *Ciona* caspases repertory is similar to vertebrates

**Figure 1:**
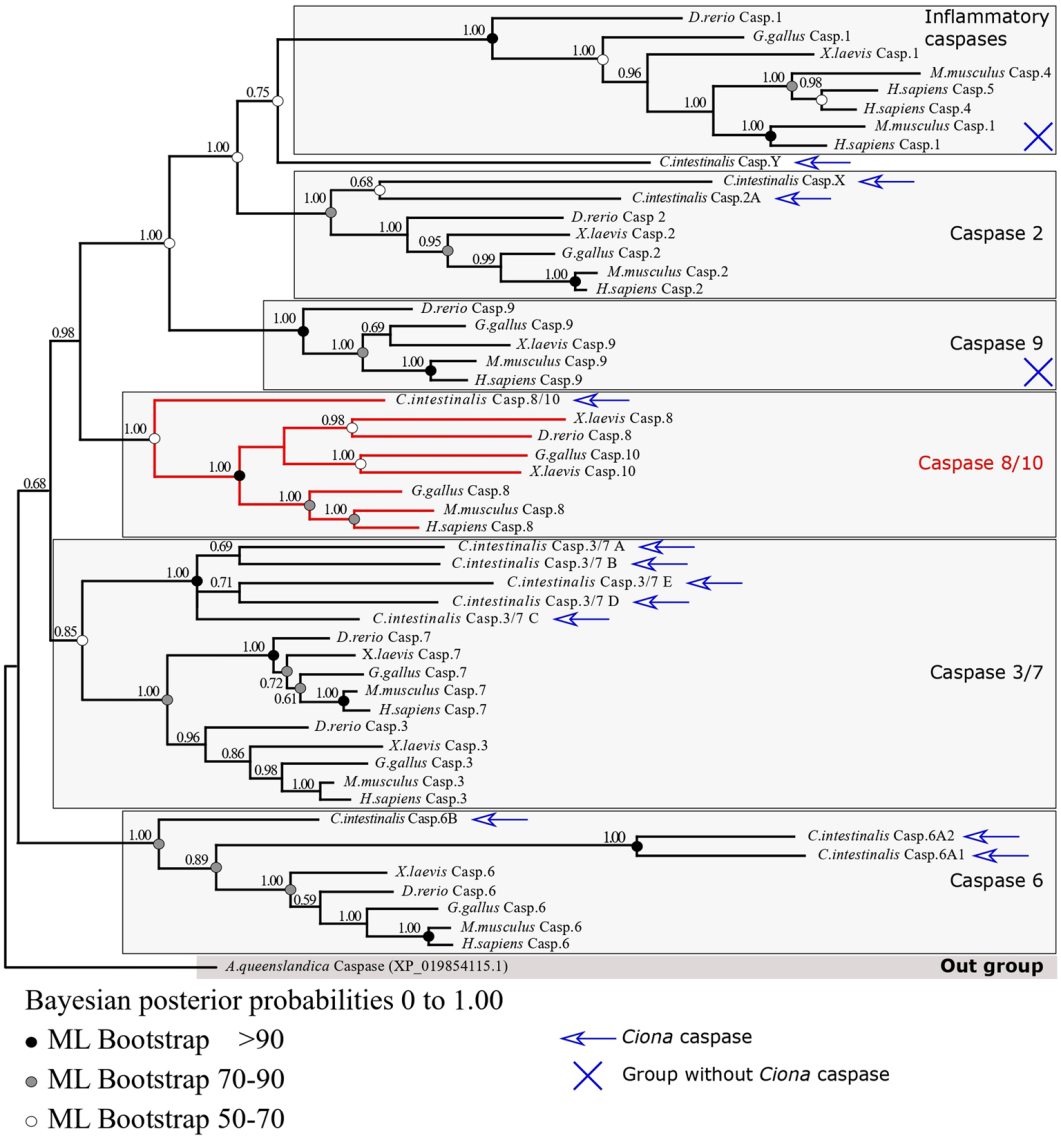
Phylogenetic analysis of caspases of *Ciona* and vertebrates obtained by Bayesian inference. Same topology made by maximum likelihood was obtained. DED-caspases are grouped together (red). CARD-caspases (caspases 2, 9 and inflammatory) formed a monophyletic clade. Executioner caspases are separated between caspase 6 clade and caspases 3 plus 7 clade. Sequences come from *Ciona* Type A genome.

We named *Ciona* caspases according to their vertebrate’s relatives (Fig. 1, Supplementary table 1, Supplementary Fig. 1) thanks to phylogenetic analysis. Our topology is similar between both Bayesian inference and maximum likelihood methods, and sequence relationships is consistent with the species one (Fig. 1). We identified six distinct clades corresponding to the groups described in vertebrates: inflammatory caspases, caspase 8 and 10 (paralogues), caspase 2, caspase 9, caspases 6 and caspases 3 and 7. All of the CARD-caspases formed a monophyletic group (posterior probability 1.00). Consistently with previous study, caspase 8 and 10 of vertebrates are paralogues and monophyletic (posterior probability 1.00) and form a clade with the orthologue relative Ci-caspase 8/10, the only *Ciona* caspase presenting the typical two DED pro-domains, characteristic of extrinsic apoptosis initiator caspases in mammals. Both the inflammatory caspases (posterior probability 1.00) and the caspases 9 group (posterior probability 1.00) contain vertebrate sequences only, suggesting the loss of *Ciona* caspase 9 as previously reported^14,28^. The caspase 2 group (posterior probability 1.00) is composed of the vertebrate sequences and *Ciona* Ci-caspase 2 and Ci-caspase X, this later do not have a CARD pro-domain. This suggested a caspase 2 duplication in *Ciona* as already reported in tunicates^14^, but one of the two paralogs having a high divergent sequence making the CARD pro-domain unidentifiable (reason why by caution we named it Ci-caspase X). Executioner caspases are separated between caspases 6 (posterior probability 1.00) and caspases 3 + 7 (posterior probability 0.85). *Ciona* presents three probable paralogs of caspases 6 (Ci-caspase 6A1, A2, and B) with a high divergence illustrated by long branch possibly explaining the decrease in nodes robustness. Caspase 3 and 7 of vertebrates are paralogues and formed a monophyletic group. Five paralogues of *Ciona* are orthologous to both caspases 3 and 7. We named them Ci-caspase 3/7 A to E. Last, Ci-caspase Y branch with the inflammatory caspases, but shows a long branch indicating high divergence making its identification unclear hence why we chose this denomination.

### Ci-casp-8/10 is expressed in the larvae

**Figure 2:**
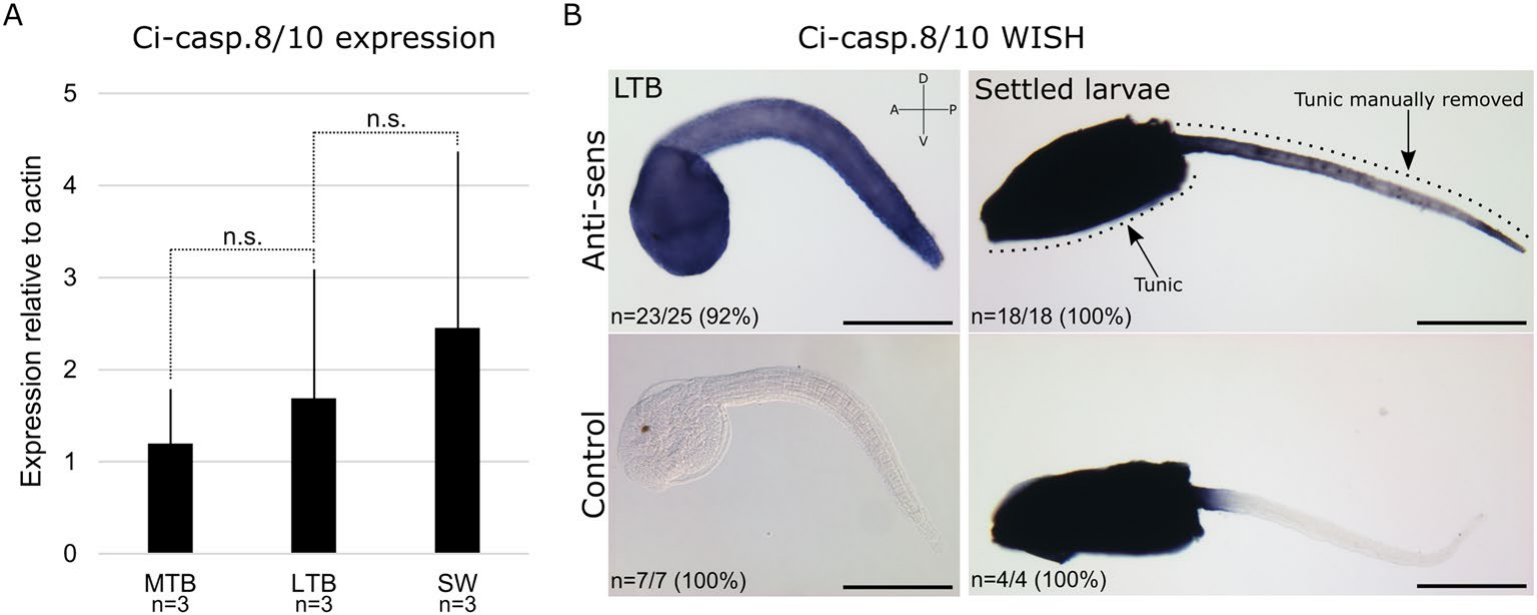
Ci-caspase 8/10 is expressed in the larvae. (**A**) real-time PCR showing expression of Ci-Casp8/10 relative to Ci-*actin* on embryonic larvae at mid tail-bub (MTB), late tail-bud (LTB), and hatching swimming larvae (SW). Error bars correspond to standard deviation. Gene expression is not statistically significant (n.s.; Wilcoxon Mann Whitney test). (**B**) whole in situ hybridisation on dechorionated LTB larvae and settled larvae. Tunic from settled larvae has been manually delated from the tail to remove the so-known background induced by it. Strong dark blue colour of the trunk results of presence of the tunic. Scale bars: (**B**) = 200 µm. Experiment were conducted on *Ciona* Type B. Orientation: *A* anterior, *P* posterior, *D* dorsal, *V* ventral.

Relative expression level of Ci-caspase 8/10 was determined by real-time PCR during embryogenesis at the mid-tail bud (MTB) and late-tail bud (LTB) stages as well as in swimming larvae (Fig. 2A). Expression level increased overtime although differences were not statistically significant. We confirmed expression of Ci-caspase 8/10 by whole in situ hybridisation (WISH) on LTB and settled larvae (Fig. 2B). Ci-caspase 8/10 expression is ubiquitous in LTB larvae with a stronger labelling at the posterior half of the tail. We manually removed the larval tunic from the tail of the settled larvae as we did previously to prevent the background generated by this tissue^30^. Settled larvae presented an expression pattern similar to the LTB, seems even reinforced at the tip of the tail, whereas remaining tunic makes impossible any expression pattern observation in the trunk due to the high background. Usually, extrinsic apoptosis also depends on TNF receptors and FADD which we identified in the *Ciona* genome (Supplementary Table 2). Interestingly, the three TNF receptors are expressed in the tail at the LTB stage, suggesting a possible role during tail regression (Supplementary Fig. 2).

### Caspase 8 inhibition delayed the tail regression

**Figure 3:**
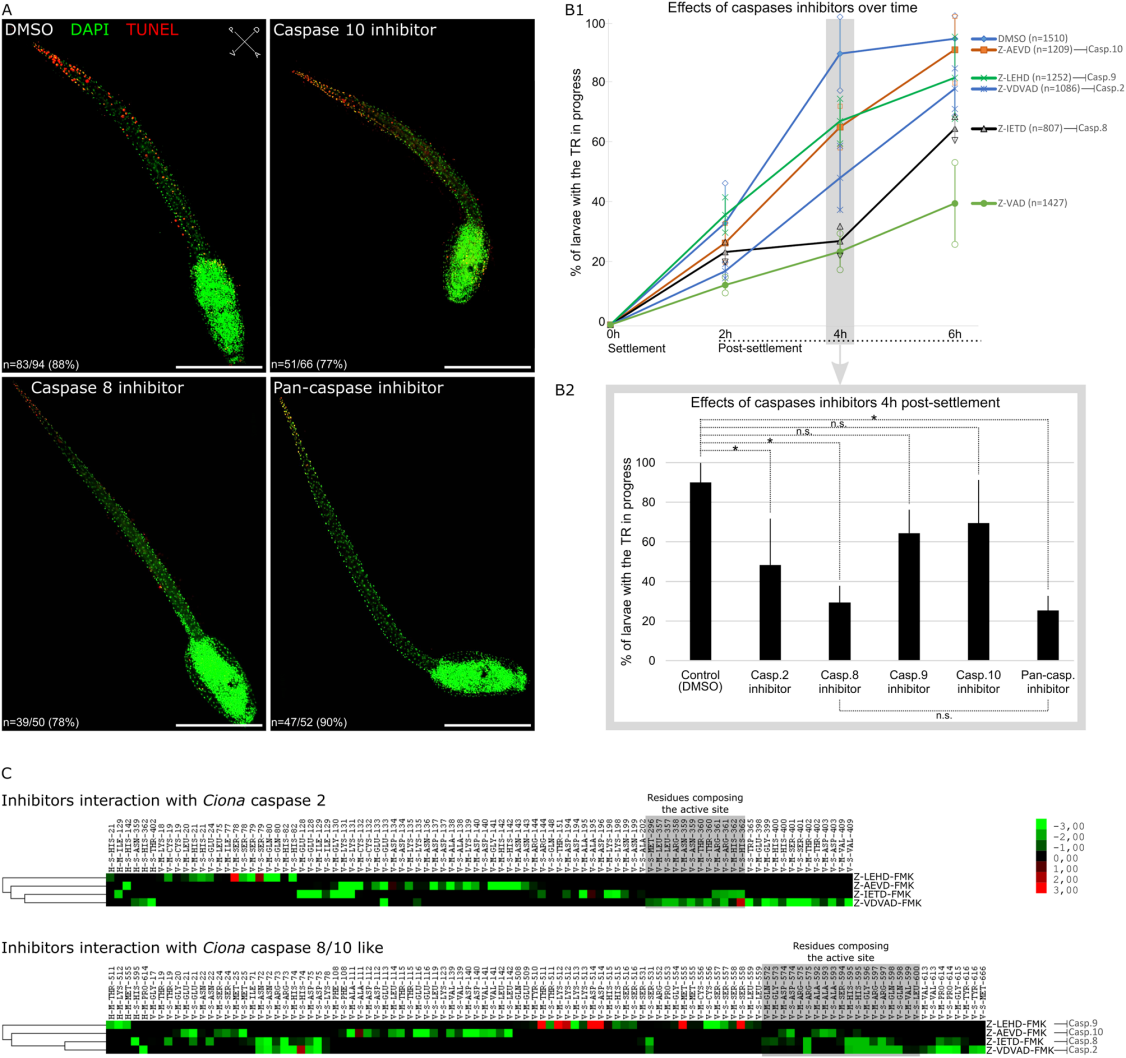
Effects of caspase inhibitors on the tail regression. (**A**) TUNEL labelling (red) counterstaining with DAPI (green) of larvae at the onset of tail regression. Apoptosis starts at the tip of the tail in control (DMSO) as in presence of the caspase 10 inhibitor. Only few apoptotic cells are detected in caspase 8 inhibitor and Z-VAD-Fmk treated larvae. (**B1**) Larvae’ proportion with the tail regression in progress overtime after settlement. (**B2**) Statistical differences of larvae’ proportion with the tail regression in progress at 4 h post-settlement from figure (**B1**). Scale bars: 200 µm. (**C**) in silico analysis of theoretical docking ability between specific caspase inhibitors and *Ciona* initiator caspases. In the profile, coloured raw indicate the preferences of the interacting residues for chemicals, with maximum red or maximum green indicating high docking probability through-black indicating low/absence of interactions. The following rows show the interactions types between the active compounds and the residues, noted H (hydrogen-bonding), E (electrostatic interaction), and V (Van der Waals interaction). Next, M and S display the main chain and the side chain of the interacting residue, respectively. Last, residue names and positions are indicating. Experiments were conducted on *Ciona* Type B. Error bars (**B1**,**B2**) correspond to standard deviation. *Wilcoxon Mann Whitney test p-value < 0.05. Orientation: *A* anterior, *P* posterior, *D* dorsal, *V* ventral.

We exposed settled larvae to the caspase 8 inhibitor (Z-IETD-Fmk), the caspase 10 inhibitor (Z-AEVD-Fmk), and the pan-caspases inhibitor (Z-VAD-Fmk; as positive control) and performed TUNEL labelling when the tail regression started in the control (DMSO). Both control and caspase 10 inhibitor treated larvae presented a similarly high number of TUNEL positive nuclei at the tip of the tail, while pan-caspase and caspase 8 inhibitors treated larvae presented only few apoptotic cells (Fig. 3A). In order to evaluate potential differences in the apoptotic profile between control (DMSO) and caspase 8 inhibitor treated larvae, we observed shape and position of TUNEL positive nuclei we detected (Supplementary Fig. 3). Consistently with several previous studies^7,26^, we deduced that tunic, epidermal, and muscle cells undergo apoptosis in the tail. In the case of caspase 8 inhibitor treatment, the same tissues were affected, indicated that the inhibitor do not modified the type of tissue undergoing apoptosis, but decrease the number of apoptotic cells. Next, we counted the number of larvae with the tail regression in progress every two hours from its beginning in the control (Fig. 3B1). The caspase 10 inhibitor had no effects on the tail regression, whereas the caspase 8 inhibitor significantly reduces the number of larvae with the tail regression in progress, especially at the 4 h’ time point (Fig. 3B2). Importantly, the caspase 8 inhibitor showed a similar result as the pan-caspase inhibitor already successfully used in *Ciona*, highlighting the strong implication of Ci-caspase 8/10 in this process^7,30^. We also treated larvae with caspase 2 inhibitor (Z-VDVAD-Fmk) which induce a moderate slowdown of tail regression, but still significant. This suggest that another pathway, based on a CARD-caspase, could be involved in apoptosis during tail regression. Conversely, caspase 9 inhibitor (Z-LEHD-Fmk) do not have significant effect, which can likely be explained by the absence of caspase 9 in *Ciona* genome.

Finally, as chemical specificity can be variable or uncertain, we tested in silico docking ability of the four specific inhibitor we used with the two *Ciona* initiator caspases, Ci-caspase 2 and Ci-caspase 8/10 (Fig. 3C). Inhibitors act by binding the active site of caspases, creating a physical blocking preventing cleavage capacity of the protease. Our modelling indicate that caspase 8 inhibitor present more binding residues affinities than caspase 10 inhibitor on active site of Ci-caspase 8/10, which could explain absence of caspase 10 inhibitor effect. This may be due to the fact that Ci-caspase 8/10 active site sequence is closest to the vertebrate caspase 8 than the caspase 10 (Supplementary Fig. 4). Ci-caspase 2 could be successfully inhibited by caspase 2 inhibitor, and seems not sensitive to caspase 8 and caspase 10 inhibitor. However, Ci-caspase 8/10 could be theoretically inhibited by caspase 2 inhibitor, maybe explaining likely the effect of this drug on tail regression. We observed that effects of treatments were transitory given that treated larvae completed the tail regression during the night post-treatment, probably due to degradation of chemicals, functional redundancy between caspases, or because the extrinsic apoptosis is likely not the only pathway implicated to trigger apoptosis during tail regression.

### Loss of function of Ci-caspase 8/10 generates an incomplete tail regression

**Figure 4:**
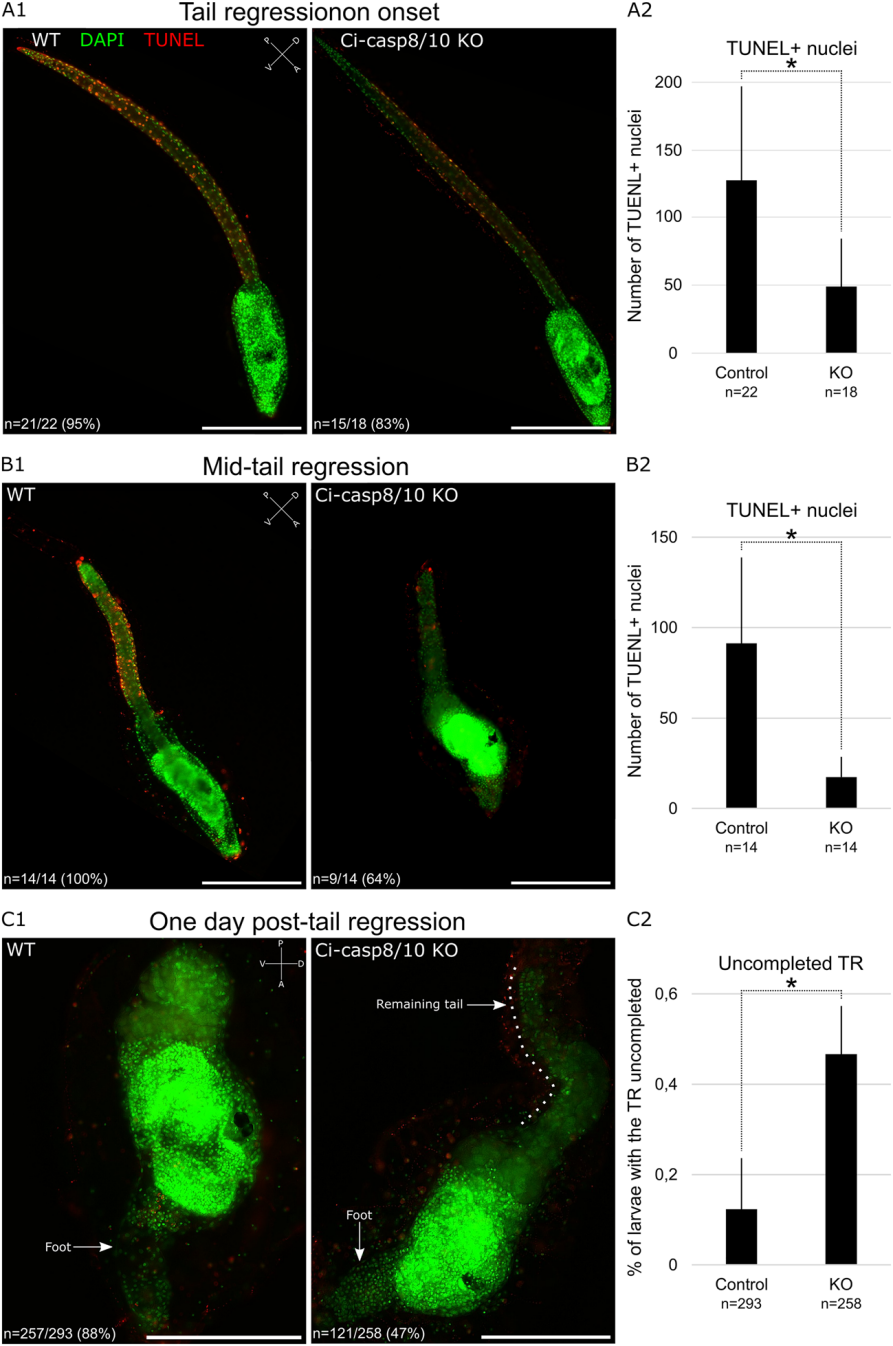
Effects of Ci-caspase 8/10 loss of function on the tail regression. (**A1**) TUNEL labelling (red) counterstaining with DAPI (green) of larvae at the onset of tail regression (TR). Ci-caspase 8/10 disrupted larvae exhibit few TUNEL + nuclei through the tail. (**A2**) the number of TUNEL + nuclei at the onset of tail regression in Ci-caspase 8/10 disrupted larvae is significantly reduced according to the control. (**B1**) TUNEL labelling of larvae at the middle of tail regression. Ci-caspase 8/10 disrupted larvae exhibit less TUNEL + nuclei than the control. (**B2**) the number of TUNEL + nuclei at the middle of tail regression in Ci-caspase 8/10 disrupted larvae is significantly reduced according to the control. (**C1**) while tail regression is over next day after settlement, a part of the Ci-caspase 8/10 disrupted larvae presents a remaining part of the tail (uncompleted TR) without any TUNEL + nuclei. (**C2**) Proportion of settled larvae having an uncompleted TR is significantly higher in Ci-caspase 8/10 disrupted larvae. Scale bars: 200 µm. WT: wild-type. Experiments were conducted on *Ciona* Type A. Error bars (A2, B2, C2) correspond to standard deviation. *Wilcoxon Mann Whitney test p-value < 0.05. Orientation: *A* anterior, *P* posterior, *D* dorsal, *V* ventral.

We injected dechorionated eggs with guide RNA against Ci-caspase 8/10. We tried two gRNA, first and second one inducing a mutation rate of 66.7% and 17%, respectively (Supplementary Fig. 5). Consequently, we pursue experiment with the gRNA number one. We performed a TUNEL labelling on settled larvae at the beginning of the tail regression and observed a significant decreasing of TUNEL-positive nuclei number in the Ci-caspase 8/10 KO larvae, especially at the tip of the tail, where the apoptosis is supposed to start (Fig. 4A). Later, at the mid-tail regression, the significant difference of TUNEL-positive nuclei number persisted and accentuated (Fig. 4B). First day post-settlement, when the tail is normally fully regressed, we observed a significant number of larvae presenting a remaining part of the tail in the Ci-caspase 8/10 KO larvae (Fig. 4C). Interestingly, this remaining tail stopped moving and did not display any apoptotic cells either, that could explain the incomplete regression. We conclude that Ci-caspase 8/10 is an initiator triggering apoptosis during the tail regression, but this caspase should not be the only one knowing that some apoptotic cells can be still detected and that the tail can undergo a part of the regression. Importantly, the significant reduction of apoptotic cells at the tail tip is consistent with the fact that the apoptotic wave starts at the most posterior part of the tail. This default in apoptosis initiation may explain secondarily the slowdown/inhibition of the regression.

According to our observations, the half tail regressed is partially accumulate into the epidermal pocket, the structure allowing tail tissues accumulation during tail regression^23,24^, which seems underdeveloped in caspase disrupted larvae. The remaining part of the tail can persist on the juvenile or disappeared, likely by progressive degradation. We did not notice any impacts on survival rate of juveniles having this phenotype, as next step of the metamorphosis went normally and the juveniles are able to feed few days after larvae settlement.

## Discussion

We showed that Ci-caspase 8/10 is orthologous to the mammalian paralogs caspase 8 and 10. Involvement of orthologous actors is a prerequisite to consider regulation pathways as homologs. Consequently, with respect to the NCCD-2018 and according to the consensus that the caspase 8 is the initiator of extrinsic apoptosis in mammals, we hypothesised that extrinsic apoptosis regulates cell death in *Ciona*. Interestingly, *Ciona*’s genome possesses four and three members of the TNF and TNFr family, respectively^28^, in addition to a FADD like protein (KH.S1574.1). Next step will be to dissect their functions and characterised their interaction with Ci-caspase 8/10 to better understand the extrinsic apoptosis in *Ciona* and draw a deeper comparison with the mammalians one.

### Ci-caspase 8/10 participates to the apoptotic event during the tail regression

Tail regression is triggered by a set of coordinate mechanisms from larvae settlement to the apoptotic-dependant tail regression^27^. Activation of the ERK at the anterior adhesive papilla in followed by JNK and b-adrenergic receptor activation, mediating the signalling among the CNS toward the tip of the tail, leading to ERK phosphorylation at the most posterior extremity of the larvae^26^. Here, due to larvae settlement, these first steps of tail regression competency acquisition likely occur normally. Ultimately, this set of events, and especially ERK phosphorylation which transduces the death-activating signal at the metamorphosis stage, lead to the activation of executioner caspases at the tip of the tail, leading to apoptosis^7,26,27^. Executioner caspase activation propagate as a wave toward the trunk, given the typical polarised apoptotic profile requires for the caudal regression of ascidians tadpoles^7,30,40^. Consequently, the posterior initiation of apoptosis is the fundamental starting point of the apoptotic wave. Here, our data show the absence of apoptotic cells at the tip of the tail of initiator Ci-caspase 8/10 disrupted larvae, indicating a default in the apoptosis initiation. Usually, executioner caspases have to be activated by initiator caspases during the course of the apoptotic signalling pathways^3,11,41^. Consequently, Ci-caspase 8/10 disruption likely prevent the activation of the executioner caspases involve in tail regression, and apoptosis initiation failure likely explain the uncompleted tail regression phenotype observed, knowing that apoptosis is required for the tail regression^7^.

The reduction of TUNEL + nuclei number and the incomplete tail regression in our Ci-caspase 8/10 disrupted larvae argue on the implication of an apoptotic pathway regulated by this initiator, potentially extrinsic apoptosis due to the presence of DED prodomain, in accordance with multiple report on caspase 8 and 10 as the specific and exclusive initiator caspase of extrinsic apoptosis in animals. However, apoptosis is not completely abolished and tail regression not fully prevented. Even if we keep in mind that the incapacity to totally blocked apoptosis in Ci-caspase 8/10 like KO larvae may be due to the mutation rate of our Crispr/CAS9 and likely reflect a mosaicism effect it suggests that Ci-caspase 8/10 may not be the only initiator regulating the polarised apoptotic wave. In mammals, several studies discovered simultaneous activations of intrinsic and extrinsic apoptosis, and that the initiation of one could trigger the activation of the second one due to interaction between their regulation systems^42–44^. Due to redundancy between intrinsic and extrinsic apoptosis we may hypothesise that the *Ciona* CARD-caspase could be responsible of an additional apoptotic signalling pathway, which works together with the extrinsic apoptosis to conduct cell death during the tail regression.

### Is the extrinsic apoptosis conserved?

DED-caspases, FADD or TNFr have been identified in most animals phyla^19,21,45–48^ and are usually considered homologs to their mammalian relatives. However, functional studies on extrinsic apoptosis outside of mammals remain drastically limited, and most of them were conducted on mammalian cultured cells. At the deuterostomians scale, activity of a DED-caspase has been shown concomitantly with detection of FAS and FADD in the ascidian *Botryllus schlosseri*^49^. The larvae of ascidian *Molgula occidentalis* present a similar apoptotic profile as *Ciona* during the tail regression^40^. Characterisation of initiator caspases triggering apoptosis in this species could bring light on the conservation of extrinsic apoptosis in chordates. In the cephalochordate *Branchiostoma belcheri tsingtauense*, two FADD proteins and a TNF are functional in human HeLa cells and seem able to form a DISC complex^18^. In the echinoderm *Holothuria leucospilota*, over-expression of FADD induces apoptosis in HEK293 human cells, suggesting conservation of its function^50^. DED-caspase of *Molgula tectiformis* (ascidian), *Paracentrotus lividus* (sea urchin), and *Strongylocentrotus purpuratus* (sea urchin) can induce apoptosis in HeLa cells^17^. Taken together, our work with previous studies on *Amphioxus* and echinoderms suggest similar activation of extrinsic apoptosis base on DED-caspases at the chordate scale and among deuterostomians.

Finally, DED-caspases from protostomians *Leucoraja erinacea* (skate), *Tubifex tubifex* (sludge worm), *Mytilus californianus* (mussel), and *Schmidted mediterranea* (planarian) are able to triggers apoptosis in human cell lines, as well as the DED-caspase from the cnidarian *Acropora millepora*^17^. Taken together, while keeping in mind that the evolutionary relationships between genes is unknow, the ability of DED-caspases to initiate apoptosis seems shared by several animals. However, the understanding of extrinsic apoptosis evolution will need further studies on animals having key phylogenetic positions.

### Supplementary Information

Supplementary Information.

## Data Availability

All data needed to evaluate the conclusions in this paper are present in the paper and/or the Supplementary Materials. Any requests can be addressed to corresponding author.
