# Supplementary material for: Extrinsic apoptosis participates to tail regression during the metamorphosis of the chordate Ciona
Source: Sci Rep. 2024 Mar 8;14:5729. doi: 10.1038/s41598-023-48411-y (PMC10923776; doi:10.1038/s41598-023-48411-y)
Supplement: Supplementary file 1 — Supplementary Information. [file 41598_2023_48411_MOESM1_ESM.doc]

**Title**

Extrinsic apoptosis participates to tail regression during the metamorphosis of the chordate *Ciona*

**Authors**

Gabriel Krasovec^1,2,3*^, Cécile Renaud^1^, Éric Quéinnec^1^, Yasunori Sasakura^2^, Jean-Philippe Chambon^4^

^*^ Authors for correspondence: GK: [gabriel.krasovec@ijm.fr](mailto:gabriel.krasovec@ijm.fr)

**Affiliations**

^1^Institut de Systématique, Evolution, Biodiversité, Sorbonne Université, Muséum National d’Histoire Naturelle, CNRS, EPHE, F-75252 Paris Cedex 05, France.

^2^Shimoda Marine Research Center, University of Tsukuba, Shizuoka, Japan.

^3^Present address : Université Paris Cité, CNRS, Institut Jacques Monod, F-75013 Paris, France.

^4^CRBM, Université de Montpellier, CNRS, 34000 Montpellier, France.

**Supplementary Table 1**: List of *Ciona* Type A caspases.

| Names | Domains | Accession numbers |
| --- | --- | --- |
| Ci-Casp.2 | CARD-P20-P10 | KH2012:KH.C4.463 |
| Ci-Casp.3/7A | P20-P10 | KH2012:KH.C12.7 |
| Ci-Casp.3/7B | P20-P10 | KH2012:KH.C12.350 |
| Ci-Casp.3/7C | P20-P10 | KH2012:KH.C12.220 |
| Ci-Casp.3/7D | P20-P10 | KH2012:KH.C12.104 |
| Ci-Casp.3/7E | P20-P10 | KH2012:KH.C8.922 |
| Ci-Casp.6A1 | P20-P10 | KH2012:KH.S1499 |
| Ci-Casp.6A2 | P20-P10 | KH2012:KH.C2.896 |
| Ci-Casp.6B | P20-P10 | KH2012:KH.C12.449 |
| Ci-Casp.8/10 | DED-P20-P10 | KH2012:KH.C8.550 |
| Ci-Casp.X | P20-P10 | KH2012:KH.C12.451 |
| Ci-Casp.Y | P20-P10 | KH2012:KH.C7.203 |

**Supplementary Table 2**: FADD and TNF receptors genes detected in the *Ciona* Type A genome and potentially involved in extrinsic apoptosis.

| Names | Accession numbers |
| --- | --- |
| TNF receptor 1 | KH.C11.251 |
| TNF receptor 2 | KH.C3.453 |
| TNF receptor 3 | KH.L116.73 |
| FADD | KH.S1574.1 |

Hs_Casp.1 MA----------------------------------------------------------

Hs_Casp.2 MAAPSA-----------------GSWSTFQH---KELMAADRGRRILGVCGMH-------

Ci_Casp.2A MKKR--------------------------------------------------------

Ci_Casp.X MD----DAPS--------------------------------------------------

Hs_Casp.3 M-----------------------------------------------------------

Hs_Casp.7 MD-----------------------------------------------------CVGWP

Ci_Casp.3/7A MER---------------------------------------------------------

Ci_Casp.3/7B MNGK--------------------------------------------------------

Ci_Casp.3/7C MDPHPADFPEPPPQPRITLRSDTVDSSVFHD--DDNLFSMDDAVEQPD-------SFDAV

Ci_Casp.3/7D MK----------------------------------------------------------

Ci_Casp.3/7E M-----------------------------------------------------------

Hs_Casp.6 MSS---------------------------------------------------------

Ci_Casp.6A1 TP----------------------------------------------------------

Ci_Casp.6A2 M-----------------------------------------------------------

Ci_Casp.6B ------------------------------------------------------------

Hs_Casp.8 MDFS------------------------------RNLYDIGEQLDSEDLASLKFLSLDYI

Hs_Casp.10 MKSQG---------QHWYSSSDKNCKVSFR----EKLLIIDSNLGVQDVENLKFLCIGLV

Ci_Casp.8/10 MDSE----------------------GEFR----LLLHQICEGLTGENVSMMKFLCKDAI

Hs_Casp.9 MDEAD-------------------------------------------------------

Ci_Casp.Y MPIEDG-VPGLGENDQIY-----AILGKFNHKEFLKFAQLEQGLGLSN------------

Hs_Casp.1 --------------------DKVLKEKRKLFIRSM-----------------GEGTINGL

Hs_Casp.2 P-----------------HHQETLKKNRVVLAKQL--------------------LLSEL

Ci_Casp.2A -------------------HRNTLIKNRVKLAKCL--------------------HLDEV

Ci_Casp.X PTDSLSNIRSTA-------------------------------------------SVHSL

Hs_Casp.3 ------------------------------------------------------------

Hs_Casp.7 PGRKW-----------------------------------------HLEKNTSCGGSSGI

Ci_Casp.3/7A ----------------------------------------------MSDETPRKPAAGDV

Ci_Casp.3/7B ---------------ENSSAGDVLKNFHP-------------------NGNQGNQTAGKV

Ci_Casp.3/7C PPNSYPSPNSSL--STSYSGGDILSMASPPL--------------PVTRITERTQTAGEV

Ci_Casp.3/7D ------------------------------------------------------------

Ci_Casp.3/7E -------------------------------------------------------QVENL

Hs_Casp.6 --------------------ASGLRRGHP------------------------AGGEENM

Ci_Casp.6A1 ------------------------------------------------------------

Ci_Casp.6A2 ------------------------------------------------------------

Ci_Casp.6B ------------------------------------------------------------

Hs_Casp.8 PQRKQEPIKDALMLFQRLQEKRMLEESNLSFLKELLFRINRLDLL-ITYLNTRKEEMERE

Hs_Casp.10 PNKKLEKSSSASDVFEHLLAEDLLSEEDPFFLAELLYII-RQKKL-LQHLNCTKEEVERL

Ci_Casp.8/10 PRSR--ELETALDFMEYFEAKGWIQEGDLGFLAEVLYRINRHDLLKLLPGVKNRRDYEEN

Hs_Casp.9 --------------------RRLLRRCRLRLVEEL--------------------QVDQL

Ci_Casp.Y ---------KIIDGIESDCANKGVEEQQFQMVRKW-------------KMRNGSKATVEV

Hs_Casp.1 LD------------------------------------ELLQT-RVLNKEEMEKVKRENA

Hs_Casp.2 LE------------------------------------HLLEK-DIITLEMRELIQAKVG

Ci_Casp.2A LQ------------------------------------YLVQC-GILTDVTVERITSKPT

Ci_Casp.X FS----------------R-------------------SDVRT-----------------

Hs_Casp.3 --------------------------------------ENTEN-----------------

Hs_Casp.7 CA-----------------SYVT---------------QMADD-----------------

Ci_Casp.3/7A LK--------IY------KSEVKTN----ATSESD---ENIDA-----------------

Ci_Casp.3/7B LM-----------------SYVDK-----------PVFEKIDR-VITPEEFLQT------

Ci_Casp.3/7C IR-----------------TYNPS-----------P--EKRDH-EVSYDELLEEDMKFSA

Ci_Casp.3/7D ------------------------------------------------------------

Ci_Casp.3/7E CQ----------------------------VCQ-----ATFDR-----------------

Hs_Casp.6 TET------D---------AFYKR--------------EMFDP-----------------

Ci_Casp.6A1 --------------------------------------EQVYR-----------------

Ci_Casp.6A2 ------------------------------------------------------------

Ci_Casp.6B --------------------------------------------SIYNSLFL--------

Hs_Casp.8 LQTPGRAQISAYRFHFCRMSWAEAN--SQCQTQSVPFWRRVDH-LLIRVMLYQISEEVSR

Hs_Casp.10 LP------------------------------------TRQRV-SLFRNLLYELSEGIDS

Ci_Casp.8/10 FQ------------------------------------AQQNNFTTYRVVCFMLADELTE

Hs_Casp.9 WD------------------------------------ALLSS-ELFRPHMIEDIQRAGS

Ci_Casp.Y LKS----KANRY----LGTTYITPEEASATPTQPSPIGQQTNN---PDTPFVILLRRVSS

Hs_Casp.1 TVMDK-TRALIDSVIP-----KGAQACQICITYIC---EEDSYLAGTLGLSADQTSGNYL

Hs_Casp.2 SFSQN-VELL------NLLPKRGPQAFDAFCE------------------ALRETKQGHL

Ci_Casp.2A RFSQN-VAFL------TCLPTRGPRAYGTFLE------------------ALRISSQEHL

Ci_Casp.X ---------------------------------------------------QSNTAINHI

Hs_Casp.3 ------------------------------------------------------------

Hs_Casp.7 ----------------------------------QGCIEEQ---------GVEDSANED-

Ci_Casp.3/7A ---------------------------------------------------IRNSSDDF-

Ci_Casp.3/7B --------------------------------------------------LTEDSIGNF-

Ci_Casp.3/7C EGVSDSVKHFYGAAIPVTCDDRVDTGAGY---------------------DTVDAGADY-

Ci_Casp.3/7D ------------------------------------------------------------

Ci_Casp.3/7E -------------------------------------------------GDSFDAGFDPI

Hs_Casp.6 ------------------------------------------------------------

Ci_Casp.6A1 -----------------------------FCLE---------------------------

Ci_Casp.6A2 ------------------------------------------------------------

Ci_Casp.6B --------------------------------------------------MSLKSAADVL

Hs_Casp.8 SELRS-FKFLLQEEISKCKLDDDMNLLDIFIEM-----EKR--------VILGEGKLDIL

Hs_Casp.10 ENLKD-MIFLLKDSLP----KTEMTSLSFLAFL-----EKQ--------GKIDEDNLTCL

Ci_Casp.8/10 GDLKI-LKRLCAGKMSKANVRRSVDVLSWLTCM-----EEE--------DLLSEGDLDFI

Hs_Casp.9 GSRRDQARQLIID-----LETRGSQALPLFIS------------------CLEDTGQDML

Ci_Casp.Y QLDENNLRMLRNNYAYCTGARNFRTALELFQYL-----EENNL----WGCGTEEEKVEIL

Hs_Casp.1 ------------------------------NMQ---------------------------

Hs_Casp.2 EDML--------------------------------------------------------

Ci_Casp.2A VDIL--------------------------------------------------------

Ci_Casp.X ------------------------------------------------------------

Hs_Casp.3 ------------------------------------------------------------

Hs_Casp.7 ------------------------------------------------------------

Ci_Casp.3/7A ------------------------------------------------------------

Ci_Casp.3/7B ------------------------------------------------------------

Ci_Casp.3/7C ------------------------------------------------------------

Ci_Casp.3/7D ------------------------------------------------------------

Ci_Casp.3/7E SV----------------------------------------------------------

Hs_Casp.6 ------------------------------------------------------------

Ci_Casp.6A1 ------------------------------------------------------------

Ci_Casp.6A2 ------------------------------------------------------------

Ci_Casp.6B MSGV-----------------------------------P--------------------

Hs_Casp.8 KRVC--------------------------------------------------------

Hs_Casp.10 EDLC--------------------------------------------------------

Ci_Casp.8/10 MSILQHLDNQKPYKLFQRVRMNDNTVTLPKNQQKSNAFQPNSHLPVASGFNSLSKQEYNH

Hs_Casp.9 ASFLR------------------------TNRQ---------------------------

Ci_Casp.Y VRKMRQLGRHDVVEIIQNFQLH-------ANQQ---------------------------

Hs_Casp.1 ----------------------------------DSQGVLSSFP----------------

Hs_Casp.2 ------------------------------------LTTLSGLQ----------------

Ci_Casp.2A ----------------------------------EKVDISNAIP----------------

Ci_Casp.X ------------------------------------------------------------

Hs_Casp.3 ------------------------------------------------------------

Hs_Casp.7 ------------------------------------------------------------

Ci_Casp.3/7A ------------------------------------------------------------

Ci_Casp.3/7B ------------------------------------------------------------

Ci_Casp.3/7C ------------------------------------------------------------

Ci_Casp.3/7D ------------------------------------------------------------

Ci_Casp.3/7E ------------------------------------------------------------

Hs_Casp.6 ------------------------------------------------------------

Ci_Casp.6A1 ------------------------------------------------------------

Ci_Casp.6A2 ------------------------------------------------------------

Ci_Casp.6B ------------------------------------------------------------

Hs_Casp.8 ------------------------------------AQINKSLLKIINDYE---------

Hs_Casp.10 ------------------------------------KTVVPKLLRNIEKYKREKAIQIV-

Ci_Casp.8/10 TSKLEPQYVSMENHRAFNGGYNQYNNYQSGQYENSSHTTTPKQSLNFHNNNREKTFQNVD

Hs_Casp.9 -----------------------------------AAKLSKPTLENL-------------

Ci_Casp.Y ----------------------------------QDVTLTHEIVRELAE-----------

Hs_Casp.1 ----------------------------AP------------------------------

Hs_Casp.2 -----------------------HVLPPLSCDYDLSLPFPVCESCPLYK--------KLR

Ci_Casp.2A -----------------------NNKPITPTTLHATITSNVHGEFVCAR--------RQP

Ci_Casp.X ------------------------NTVQSPTSTKAATSYQINLHQ---------------

Hs_Casp.3 ------------------------------------------------------------

Hs_Casp.7 ------------------------------------------------------------

Ci_Casp.3/7A ------------------------------------------------------------

Ci_Casp.3/7B ------------------------------------------------------------

Ci_Casp.3/7C ------------------------------------------------------------

Ci_Casp.3/7D ------------------------------------------------------------

Ci_Casp.3/7E ------------------------------------------------------------

Hs_Casp.6 ------------------------------------------------------------

Ci_Casp.6A1 ------------------------------------------------------------

Ci_Casp.6A2 ------------------------------------------------------------

Ci_Casp.6B ----------------------------TP------------------------------

Hs_Casp.8 ------------------------------------------------------------

Hs_Casp.10 ----------------------------TPPVDKEAESYQGEEELVS------QTDVKTF

Ci_Casp.8/10 ESDTTWKQNTTSGSSTAPYSYSYPHRQNEPMTENPSFYYTGNNSTTMPSVPIQNAGSSET

Hs_Casp.9 ----------------------------TPVVLRPEIRKP--------------------

Ci_Casp.Y -----------------------RSNSSTPFVENVDATY---------------------

Hs_Casp.1 -QAVQDNP-----------------------AMPTSSGSE-------GNVKLCSL-----

Hs_Casp.2 LSTDTVEH-------------------------SLDNKDG--------------------

Ci_Casp.2A MEIGKCED-------------------------KNDAKDG--------------------

Ci_Casp.X ---------------------------------GMDLFDG------------PTV-----

Hs_Casp.3 ---------------------------------SVDSKSI--------KNLEPKI-----

Hs_Casp.7 ---------------------------------SVDAKPD-------RSSFVPSL-----

Ci_Casp.3/7A ---------------------------------YKDDDDD-------GKGLVPTV-----

Ci_Casp.3/7B ---------------------------------GDTVDGD-------ESHKIPEW-----

Ci_Casp.3/7C ---------------------------------GSDAGPD-------NSDTGPPL-----

Ci_Casp.3/7D ------------------------------------------------------------

Ci_Casp.3/7E ------------------------------------------------------------

Hs_Casp.6 ------------------------------------------------------------

Ci_Casp.6A1 ------------------------------------------------------------

Ci_Casp.6A2 ------------------------------------------------------------

Ci_Casp.6B -HTNENNN-----------------------VGQSEVDAL-------GSGETPRI-----

Hs_Casp.8 -EFSKGEELCG----------------------VMTISDS--------------------

Hs_Casp.10 LEALPQESWQN--------------------KHAGSNGNR-------ATNGAPSL-----

Ci_Casp.8/10 LELNRQQDYSGGQSNQFPYPTQYSGVSSNGNPIVGTTNNV-------GCDGTPMVRDYPN

Hs_Casp.9 -EVLRPET-----------------------PRPVDIGSG-------GFGDVGAL-----

Ci_Casp.Y VSVPRTES-----------------------PVPASISSSYLGNMALGDQSDPISEDICS

Hs_Casp.1 ------------EEAQRIWKQKS------------AEIYPIMDKSSRTRLALIICNEEF-

Hs_Casp.2 ----PVCLQVKPCTPEFYQTHFQ-------------LAYRLQ--SRPRGLALVLSNVHFT

Ci_Casp.2A ----HNRLRVQNSTVAFFNETYN-------------SGYKMT--TKSRGYALIVCIGRFN

Ci_Casp.X -----SDLTVRKSTHQHLMQHTDC--------------YQMTMRGRPKGAALIISVEKFH

Hs_Casp.3 -----IHGSESMDSGISLDNS-----------------YKMD--YPEMGLCIIINNKNFH

Hs_Casp.7 -----FSKKKKNVTMRSIKTTRDRVP---------TYQYNMN--FEKLGKCIIINNKNFD

Ci_Casp.3/7A -------------------------------LRQHMLEYYMK--NERRGTFVVFNQENFD

Ci_Casp.3/7B ------------------SETTD-------------FKYKMN--HKERGCFMIFNQEVYD

Ci_Casp.3/7C ----PYKQKSKVIEPEAIAVNRE-------------FQYPMN--HKQRGCCIIFNQKHFD

Ci_Casp.3/7D --------------------------------------YTIT--------------QKFD

Ci_Casp.3/7E ----PIRHPDRTTVRRELKDGDDKIEPSVWDVKFDYFEYNMS--FPKRGIFLIFDQENFD

Hs_Casp.6 -----------------------------------AEKYKMD--HRRRGIALIFNHERFF

Ci_Casp.6A1 ----KFYKNNRKMQEEWIDDC-----------------YDINY-SGKIGIAAVFLLKKRN

Ci_Casp.6A2 --------------EISINES-----------------YKIDYSHEKIGLAVVFLSEMKS

Ci_Casp.6B -----YSLPDVTKMASSEVEC-----------------YQMD--RESRGLALIINNENFH

Hs_Casp.8 -----------PREQDSESQTLD-------------KVYQMK--SKPRGYCLIINNHNFA

Hs_Casp.10 -----VSRGMQGASANTLNSETSTKR---------AAVYRMN--RNHRGLCVIVNNHSF-

Ci_Casp.8/10 GFETPPPRAQPKAPVSEIKDTSTMGI---------LDKYPME----RRGYCLIINNENFE

Hs_Casp.9 ---------------ESLRGNAD-------------LAYILS--MEPCGHCLIINNVNFC

Ci_Casp.Y -FQGNNVQVLESDDMSYVTHFSK------------VGVYTVRS-KEPKGHVLILNNYEGF

* :

Hs_Casp.1 ------------------------DSIPRRTGAEVDITGMTMLLQNLGYS-VDVKK-NLT

Hs_Casp.2 GE----------------------KELEFRSGGDVDHSTLVTLFKLLGYD-VHVLC-DQT

Ci_Casp.2A AG----------------------VRLPDRQGTDADKKNLLAIFEQINYK-TILVE-NCN

Ci_Casp.X PE----------------------SDLLNREGSEKDRVRLELVLQQIGFQ-CYVLI-NGT

Hs_Casp.3 KS----------------------TGMTSRSGTDVDAANLRETFRNLKYE-VRNKN-DLT

Hs_Casp.7 KV----------------------TGMGVRNGTDKDAEALFKCFRSLGFD-VIVYN-DCS

Ci_Casp.3/7A RM------------------------RHPRKGSGVDVEMLVKSANKLGFEDVRVLK-NQT

Ci_Casp.3/7B AKTS--------------------LNLGERIGSSTDARNLASAAAYLGFEYIQVFN-NLT

Ci_Casp.3/7C AH----------------------LRMDTREGTNHDAENIKHSMEMLGFEHVRIIK-DST

Ci_Casp.3/7D KH----------------------LKLSERTGSDKDAAALLKTAELLGFE-TEEYL-DLE

Ci_Casp.3/7E ------------------------YGLQQRVGSKLDRIVLEETAIKLGFT-PNVCH-DYT

Hs_Casp.6 WH----------------------LTLPERRGTCADRDNLTRRFSDLGFE-VKCFN-DLK

Ci_Casp.6A1 DATTNEL-----------------ENEKIEQALQQDFDDFTELFENMQLD-VMKFGEKMK

Ci_Casp.6A2 ------------------------DSPETKKALLEDYEDFKKLFADMRFD-VKSYSGEMK

Ci_Casp.6B HA----------------------TRMNKRSGTDVDARNLSRIFKKLGFD-VQVYK-DLS

Hs_Casp.8 KAREK---------------VPKLHSIRDRNGTHLDAGALTTTFEELHFE-IKPHH-DCT

Hs_Casp.10 ------------------------TSLKDRQGTHKDAEILSHVFQWLGFT-VHIHN-NVT

Ci_Casp.8/10 RQVNEDEIQRRLQMTERDFPVPN-VGLKDRTGSGNDTERLENLFKEFGFI-LDVRK-DLD

Hs_Casp.9 RE----------------------SGLRTRTGSNIDCEKLRRRFSSPHFM-VEVKG-DLT

Ci_Casp.Y AW----------------------GPDHDRKGAKRDGELMKQLWEGFQCK-VIVKE-NRT

. . * : .

Hs_Casp.1 ASDMTTELEAFAHRP-EHKTSDSTFLVFMSHGIR-------EGICGKKHSEQVPDILQLN

Hs_Casp.2 AQEMQEKLQNFAQLP-AHRVTDSCIVALLSHGVE-------GAIYGVDGK-----LLQLQ

Ci_Casp.2A KFEMERRVKQFAQME-EHRKCDSCAVAILSHGSK-------TDIYASDGR-----SIPIE

Ci_Casp.X AEQIVSTLQTFAELE-EHYYNSCSLVAAMSHGDA-------GCFYGSDGV-----SVAID

Hs_Casp.3 REEIVELMRDVSKED--HSKRSSFVCVLLSHGEE-------GIIFGTNG------PVDLK

Hs_Casp.7 CAKMQDLLKKASEED--HTNAACFACILLSHGEE-------NVIYGKDG------VTPIK

Ci_Casp.3/7A TDEIRDHLQELSYQD--HSNCDCFVCVVLSHGES------DGVLYTKDG------DIHLK

Ci_Casp.3/7B RSKILKWLKDVAAAS--HESYDCFGCAILTHGDK-------NNDY----------IMNIT

Ci_Casp.3/7C KVEIINWIAAVSKAD--HSNYDCFACVILTHGGD------KDVLYARDD------KMELK

Ci_Casp.3/7D RKQIRRKLYELSEMD--HGDHDCFACAILTHGGK------DNILYSHDD------EMKLK

Ci_Casp.3/7E KDDVYAELRKISQMN--HSQYDCFACAILTHGEE------DEMVYAKDD------SMKLK

Hs_Casp.6 AEELLLKIHEVSTVS--HADADCFVCVFLSHGEG-------NHIYAYDA------KIEIQ

Ci_Casp.6A1 FSEAVANLENVSKKLKSENNYSCFICMFIGHGSD-------GCIKATDGK-----LDIHE

Ci_Casp.6A2 FSTVVENLEAVLEEIQRNPLISCFICMFIGHGGP-------GFINTADGK-----LDIHE

Ci_Casp.6B CISMLEVINEVSTMD--HTGNDCCFVAFLSHGDD-------GCVYGTDG------IVQIK

Hs_Casp.8 VEQIYEILKIYQLMD--HSNMDCFICCILSHGDK-------GIIYGTDGQ-----EAPIY

Hs_Casp.10 KVEMEMVLQKQKCNP-AHADGDCFVFCILTHGRF-------GAVYSSDEA-----LIPIR

Ci_Casp.8/10 QREMEKVIQEYTKKD--HSDKDCFVCVVMSHGLS-------GCVYGVDGL-----SLSTS

Hs_Casp.9 AKKMVLALLELAQQD--HGALDCCVVVILSHGCQASHLQFPGAVYGTDGC-----PVSVE

Ci_Casp.Y AGAMFKFLNDFSRSS-FHQSCDFCAVVIMSHGGLVDN---RDVFYGVDCR-----TIAAN

: . : **

Hs_Casp.1 AIFNMLNTKNCPSLKDKPKVIIIQACRGDSPGV-VWFKDSVGVSGNL----SLPTTEEFE

Hs_Casp.2 EVFQLFDNANCPSLQNKPKMFFIQACRGDETDRGVDQQDGKNHAGSPGCEESDAGK----

Ci_Casp.2A SLIKMFDNVNCPPLRNKPKIFFIQCCRGNNMDQGIDATDGPPTLNSETSDSYNSTTHNIT

Ci_Casp.X TVVNFFSNQNCHSLQKKPKIFLFQACQGDEYDMGVDEVDGPVQAPVGDVD--NTSTSSSN

Hs_Casp.3 KITNFFRGDRCRSLTGKPKLFIIQACRGTELDCGIETDSGVDDDM---------------

Hs_Casp.7 DLTAHFRGDRCKTLLEKPKLFFIQACRGTELDDGIQADSGPINDTD--------------

Ci_Casp.3/7A EILDSFKASRCPSLAGKPKLFFIQACRGEKRSTPVEIEYKFDV--------TDSTPPEEQ

Ci_Casp.3/7B DFTNPFTADKCPTLAGKPKLFFVQACRGHKRDTAVPFTLNVASTI---------------

Ci_Casp.3/7C DFMQPFRGDNCPSLATKPKLFFIQACRGFKLAEPVKVRPVQCDSLNY--DTTDGGSATVD

Ci_Casp.3/7D DFTTPFEADKCRSLASKPKLFFVQACRGDLLDRGAKVIHLKQSTGDV----LDRNPSKVE

Ci_Casp.3/7E TLISRVSATECPSLAGKPKLFFVQACRGKEISQPAICTTACLKRQQV----QSDSIPDVD

Hs_Casp.6 TLTGLFKGDKCHSLVGKPKIFIIQACRGNQHDVPVIPLDVVDNQTEK----LDTNITEVD

Ci_Casp.6A1 NILAKFMTHKC--LEGKPKIFIFQACRGTKKNVKSDSSLSTLCDKEI-------------

Ci_Casp.6A2 NILSKFMPQDC--LKGKPKIFIFQTCRGTRHNFDSLACSGTLTVPTV-------------

Ci_Casp.6B KIVDQFRGDVCPSLAGKPKIFLFQACRGTLHETSVQLSVDVVDGE-------DQPCVEVD

Hs_Casp.8 ELTSQFTGLKCPSLAGKPKVFFIQACQGDNYQKGIPVETDSEEQPYL----------EMD

Hs_Casp.10 EIMSHFTALQCPRLAEKPKLFFIQACQGEEIQPSVSIEADALNPEQA-------------

Ci_Casp.8/10 KISKSFRPGMCSSLTGKPKIFFFQACQGDRTMDGHSAPVDDVESDGA-------------

Hs_Casp.9 KIVNIFNGTSCPSLGGKPKLFFIQACGGEQKDHGFEVASTSPEDESPGSN-PEPDATPFQ

Ci_Casp.Y DVLALFKNTHSKYLIGRPKLIFFQCCRGGSSSRGVPFVDAADAVNRL-----LPPSDQTD

. . . * :**:::.* * *

Hs_Casp.1 ---------DDAIKKAHIEKDFIAFCSSTPDNVSW----RHPTMGSVFIGRLIEHM----

Hs_Casp.2 --------EKLPKMRLPTRSDMICGYACLKGTAAM----RNTKRGSWYIEALAQVF----

Ci_Casp.2A ---TDVDIISTASKRLPTSSDMIIGYATLQGNAAL----RNTRHGSWYIQVLVKKI----

Ci_Casp.X ---------DHIRNKLPQKSDMLIGQATMKGFAAM----RNTKHGSWYIQAFVRVL----

Hs_Casp.3 -----------ACHKIPVEADFLYAYSTAPGYYSW----RNSKDGSWFIQSLCAML----

Hs_Casp.7 ---------ANPRYKIPVEADFLFAYSTVPGYYSW----RSPGRGSWFVQALCSIL----

Ci_Casp.3/7A ---------TTTVFTIPAEADFLIAHATPEEFCAW----RNRREGSRFIKALCGCL----

Ci_Casp.3/7B --------EKTELKLIPCQADFLIASSTPEGYSSW----RNKANGSWFIQALYLCI----

Ci_Casp.3/7C -----------EFATIPAEADFLIAQSTVPKYYSW----RNKSNGSIFIQALCLTF----

Ci_Casp.3/7D ------------SYTIPVQADFLISQATAPDYYAW----RSSDKGSIFIQTLCSVF----

Ci_Casp.3/7E ---------SEETPKIPAEADILVAYSTQPGQVSV----RNEYTGSVFVQTMCAAL----

Hs_Casp.6 ---------AASVYTLPAGADFLMCYSVAEGYYSH----RETVNGSWYIQDLCEML----

Ci_Casp.6A1 ---------------KADGSDVLLCYAAWSGYVSYALTTNEYERGTQYLQDLHKAL----

Ci_Casp.6A2 --------------LNPEGSDMLLCYASWQGFVSYC--VNTKGKGTCYLQELHSML----

Ci_Casp.6B ---------AAPVPTLPSGSDYLFCYSVAEGYYSH----RDTLYGSWFIQDFTDVMEQLV

Hs_Casp.8 -------LSSPQTRYIPDEADFLLGMATVNNCVSY----RNPAEGTWYIQSLCQSL----

Hs_Casp.10 --------PTSLQDSIPAEADFLLGLATVPGYVSF----RHVEEGSWYIQSLCNHL----

Ci_Casp.8/10 --------ASHGRQVLPSESDFLIGHSTVPGYLSY----RSRTDGSWFISTLVDSL----

Hs_Casp.9 EGLRTFD-QLDAISSLPTPSDIFVSYSTFPGFVSW----RDPKSGSWYVETLDDIF----

Ci_Casp.Y ---------GIDKTTLPHMSDILVAYSTLEGDMSF----RNEATGSWFINAIATVF----

* : : : . *: :: : :

Hs_Casp.1 ---QEY-ACSC-DVEEIFRKVRFSFEQP----------DGRAQMPTTERVTLTRCFYLFP

Hs_Casp.2 ---SER-ACDM-HVADMLVKVNALIKDR-EGYAPGTEFHRCKEMSEYCS-TLCRHLYLFP

Ci_Casp.2A ---AQH-AHEM-DLLEILTLVNSAIKSK-EGHSTDFDSQGVKEMSEFHS-TLCKKLFFYP

Ci_Casp.X ---ARH-ACDT-DLLDMMTKVNNILKHK-EGWCPGSVYHSCTYLHILPA-KLNNHHFCCP

Hs_Casp.3 ---KQY-ADKL-EFMHILTRVNRKVATEFESFSFDATFHAKKQIPCIVS-MLTKELYFYH

Hs_Casp.7 ---EEH-GKDL-EIMQILTRVNDRVARHFESQSDDPHFHEKKQIPCVVS-MLTKELYFSQ

Ci_Casp.3/7A ---DRYAGDDI-ELLQILTVVNHHVSHGWDGEVY--KYEKSKQIPSITT-QLTAQLFFTK

Ci_Casp.3/7B ---QRY--PSL-EIMQIMTRVNRMVALHFENTNTE-SPDIYKQIPSICT-RLTGELYFHK

Ci_Casp.3/7C ---NLF-GEKQ-EIMQMMTKVNRMVAYDYESASKDPKMNQKKQIPSITT-QLTAELYFPK

Ci_Casp.3/7D ---ERY-SDEM-DIMKILTRVNRVVAFNFESWTQRPDMNHMKQIPSITS-QLTAELFIKK

Ci_Casp.3/7E ---KQR-GGEL-ELVQLLTRVNRNIALTFETNMCSPKFDKKKQMPSIVS-QLTAELYFRP

Hs_Casp.6 ---GKY-GSSL-EFTELLTLVNRKVSQRRVDFCKDPSAIGKKQVPCFAS-MLTKKLHFFP

Ci_Casp.6A1 ---RMF-TKP--DKPQSISSTN--------------------------------------

Ci_Casp.6A2 ---RKF-ADGTNHLIDILTVLNRQVSSS--PIMNSQRTDYFYQMPSFES-SLRKKLFLLV

Ci_Casp.6B LNKGKQ-QDKV-DFIDVLTVVNRKVSARRVERSYSQQALGKKQMPCYLS-MLTKKLYLTP

Hs_Casp.8 ---RERCPRGD-DILTILTEVNYEVSNK------DDKKNMGKQMPQPTF-TLRKKLVFPS

Hs_Casp.10 ---KKLVPRHE-DILSILTAVNDDVSRRVD------KQGTKKQMPQPAF-TLRKKLVFPV

Ci_Casp.8/10 ---EKY-HEKE-DLLSIMINVNQEMASK-----------PYKQMPMPIA-TLRKKVFFSK

Hs_Casp.9 ---EQW-AHSE-DLQSLLLRVANAVSVK----------GIYKQMPGCFN-FLRKKLFFKT

Ci_Casp.Y ---SKK-ARTE-HVVDMLTEVGRGVSHRAANTPDNMATHRCKEMSEYKS-TLRNKLYLFP

. :

Hs_Casp.1 ------------------------------------------------GH----------

Hs_Casp.2 ------------------------------------------------GHPPT-------

Ci_Casp.2A ------------------------------------------------GIVQTEKE----

Ci_Casp.X ------------------------------------------------------------

Hs_Casp.3 ------------------------------------------------------------

Hs_Casp.7 ------------------------------------------------------------

Ci_Casp.3/7A KSKNKICQEK---------PQGTIKENENANAQANDDFIAHEGQ-------ETEKEISR-

Ci_Casp.3/7B PGSTVVESH--------------ISNNDASN--AYDQSIAIETTTEERTYKRSKSTF---

Ci_Casp.3/7C KREGHPSASEYSVNQQVTSPR-QLQHNQVTARCEYDQYSSHTVRASDNAFVETTTQFSRL

Ci_Casp.3/7D PKRNTNPST---------------------------------------------------

Ci_Casp.3/7E -KDTVS------------------------------------------------------

Hs_Casp.6 KSN---------------------------------------------------------

Ci_Casp.6A1 ------------------------------------------------------------

Ci_Casp.6A2 DQR---------------MPE-----------------TAPTGDRGDSGDFQS-------

Ci_Casp.6B KK----------------------------------------------------------

Hs_Casp.8 D-----------------------------------------------------------

Hs_Casp.10 ------------------------------------------------------------

Ci_Casp.8/10 QNSNSSSNQN------CNTPEGCL------------------------------------

Hs_Casp.9 S-----------------------------------------------------------

Ci_Casp.Y ------------------------------------------------GFPKAEEE----

Hs_Casp.1 ------------------------------------------------------------

Hs_Casp.2 ------------------------------------------------------------

Ci_Casp.2A ------------------------------------------------------------

Ci_Casp.X ------------------------------------------------------------

Hs_Casp.3 ------------------------------------------------------------

Hs_Casp.7 ------------------------------------------------------------

Ci_Casp.3/7A --------------------------------------PIPSP-----------------

Ci_Casp.3/7B ------------------------------------------------------------

Ci_Casp.3/7C AMQAGDASRLEEAYMDPRTQGAAANPPSHASVRQRDLFPRPPPLPTKQRQTRRSNDSSHR

Ci_Casp.3/7D ------------------------------------------------------------

Ci_Casp.3/7E ------------------------------------------------------------

Hs_Casp.6 ------------------------------------------------------------

Ci_Casp.6A1 ------------------------------------------------------------

Ci_Casp.6A2 ------------------------------------------------------------

Ci_Casp.6B ------------------------------------------------------------

Hs_Casp.8 ------------------------------------------------------------

Hs_Casp.10 ------------------------------------------PL----------------

Ci_Casp.8/10 ------------------------------------------------------------

Hs_Casp.9 ------------------------------------------------------------

Ci_Casp.Y ------------------------------------------------------------

Hs_Casp.1 ------------------------------------------------------------

Hs_Casp.2 ------------------------------------------------------------

Ci_Casp.2A ------------------------------------------------------------

Ci_Casp.X ------------------------------------------------------------

Hs_Casp.3 ------------------------------------------------------------

Hs_Casp.7 ------------------------------------------------------------

Ci_Casp.3/7A ------------------------------------------------------------

Ci_Casp.3/7B ----------------------------------------------------LQRIRKSF

Ci_Casp.3/7C RSGDYTTRRPPQEVYSSPTGSPRDVTPHYVTGYDAPPRRSSHETSPYGSQHNVARRRPSY

Ci_Casp.3/7D ------------------------------------------------------------

Ci_Casp.3/7E ------------------------------------------------------------

Hs_Casp.6 ------------------------------------------------------------

Ci_Casp.6A1 ------------------------------------------------------------

Ci_Casp.6A2 ------------------------------------------------------------

Ci_Casp.6B ------------------------------------------------------------

Hs_Casp.8 ------------------------------------------------------------

Hs_Casp.10 ------------------------------------------------------------

Ci_Casp.8/10 ------------------------------------------------------------

Hs_Casp.9 ------------------------------------------------------------

Ci_Casp.Y ------------------------------------------------------------

Hs_Casp.1 ------------------------------------------------------------

Hs_Casp.2 ------------------------------------------------------------

Ci_Casp.2A ------------------------------------------------------------

Ci_Casp.X ------------------------------------------------------------

Hs_Casp.3 ------------------------------------------------------------

Hs_Casp.7 ------------------------------------------------------------

Ci_Casp.3/7A ---------------------------------------RHK------------------

Ci_Casp.3/7B ---------------------------------------RRGKKTKT-------------

Ci_Casp.3/7C ETSFPAGSRAIPAVTLRKQSLPVSSMYSSSADSHLLTPNRHLHPSETVPARLSVSPNAAR

Ci_Casp.3/7D ------------------------------------------------------------

Ci_Casp.3/7E ------------------------------------------------------------

Hs_Casp.6 ------------------------------------------------------------

Ci_Casp.6A1 ------------------------------------------------------------

Ci_Casp.6A2 ------------------------------------------------------------

Ci_Casp.6B ------------------------------------------------------------

Hs_Casp.8 ------------------------------------------------------------

Hs_Casp.10 ------------------------------------------------------------

Ci_Casp.8/10 ------------------------------------------------------------

Hs_Casp.9 ------------------------------------------------------------

Ci_Casp.Y ------------------------------------------------------------

Hs_Casp.1 ---------------------------------------

Hs_Casp.2 ---------------------------------------

Ci_Casp.2A ---------------------------------------

Ci_Casp.X ------------------------------------FQI

Hs_Casp.3 ---------------------------------------

Hs_Casp.7 ---------------------------------------

Ci_Casp.3/7A ---------------------------------KTETQV

Ci_Casp.3/7B --------------------------------GRHSATF

Ci_Casp.3/7C NSVNRRSLPLGTDPKPQAVSSWGSGINRAGLRSTKQTEV

Ci_Casp.3/7D ---------------------------------------

Ci_Casp.3/7E ---------------------------------------

Hs_Casp.6 ---------------------------------------

Ci_Casp.6A1 ---------------------------------------

Ci_Casp.6A2 --------------------------------SQLNVNV

Ci_Casp.6B ---------------------------------------

Hs_Casp.8 ---------------------------------------

Hs_Casp.10 ----------------------------------DALSL

Ci_Casp.8/10 ---------------------------------------

Hs_Casp.9 ---------------------------------------

Ci_Casp.Y ---------------------------------DNSLFM

**Supplementary Figure 1**: Alignment of *Ciona* Type A and human caspases.


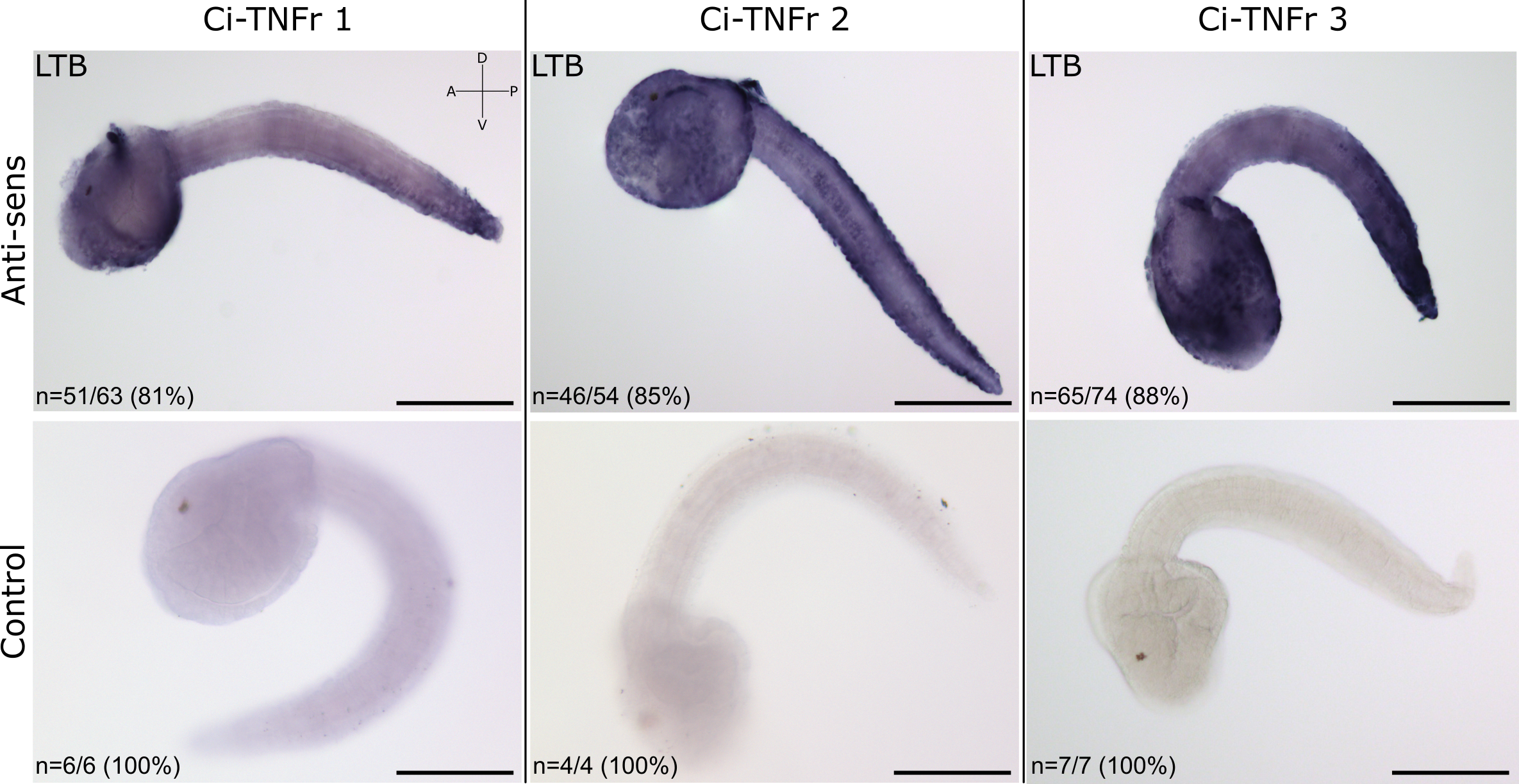


**Supplementary Figure 2**: TNF receptors are expressed in the tail during development. TNFr 1 is mainly expressed at the tip of the tail in epidermal cells. TNFr 1 and 2 are expressed in the trunk and the tail with a stronger expressed at the posterior extremity. Experiment were conducted on *Ciona* Type B. Orientation: A, anterior; P, posterior; D, dorsal; V, ventral. Scale bars = 200 µm.


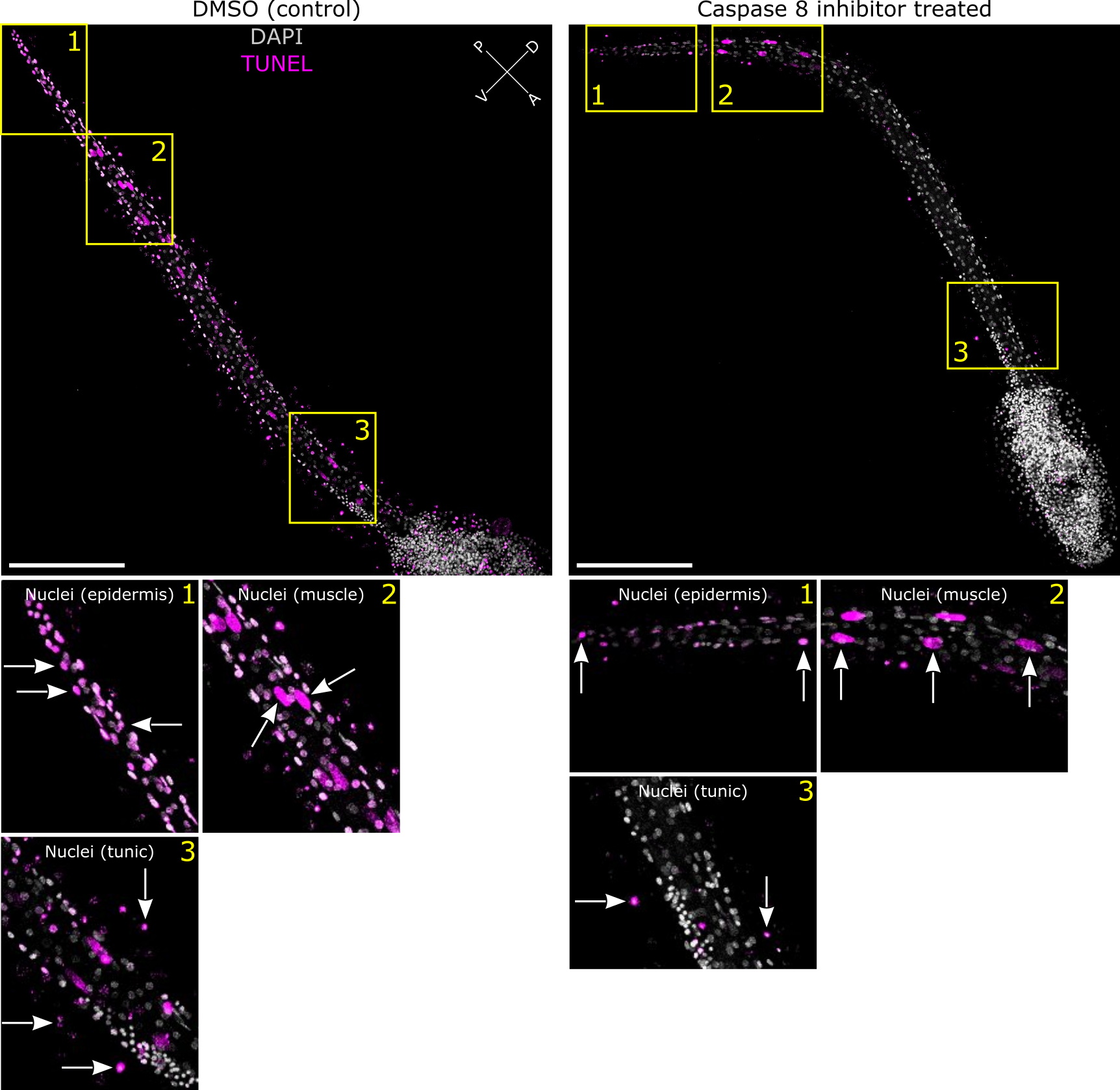


**Supplementary Figure 3**: Z-projection of *Ciona* type B labelled by TUNEL and imaged by confocal microscopy. Experiment were conducted on *Ciona* Type B. Orientation: A, anterior; P, posterior; D, dorsal; V, ventral. Scale bars = 200 µm.


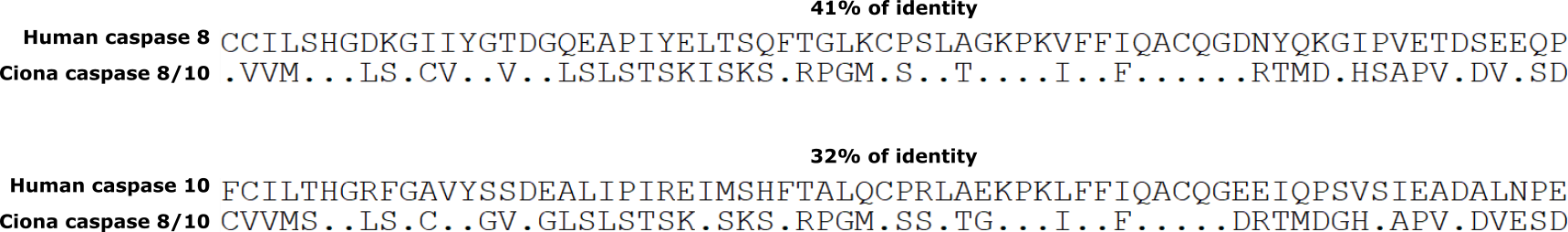


**Supplemental figure 4**: Alignments of active sites of human caspases 8 and 10 with Ci-caspase 8/10. Dots represent same amino acids between *Ciona* Type A and human caspases.


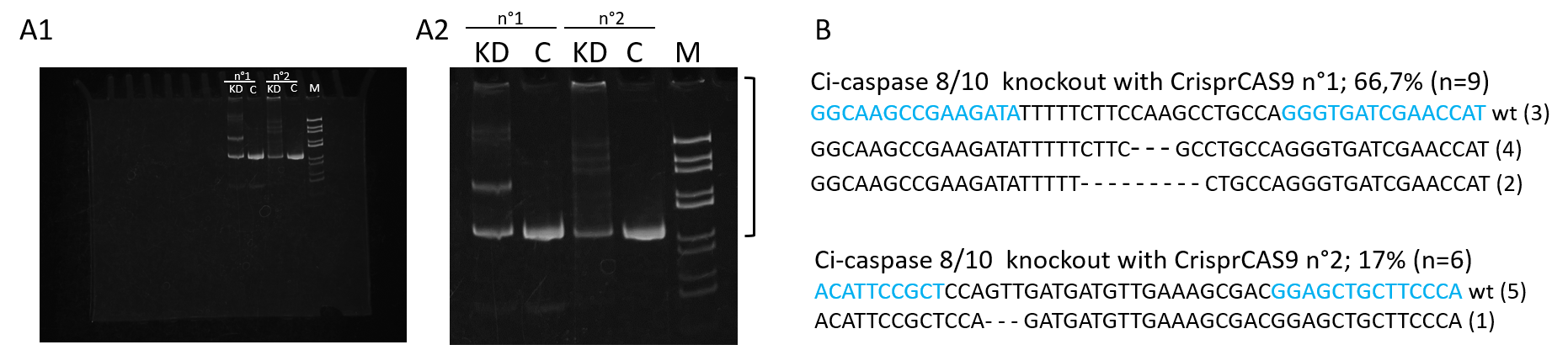


**Supplemental figure 5**: **A**, heteroduplex mobility shift assay showing heterogeneous mutated DNA sequence in the amplified Ci-Casp8/10 amplicons from *Ciona* type A (A1). Heterogeneous mutated DNA is highlighted (*bracket*) in gel magnification (A2). M: marker (pBluescript digested by HaeIII); C: Control lane without mutated Ci-Casp8/10; KD: the lane from CrisprCAS9 mix introduced animals. **B**, sequencing of Ci-caspase 8/10 genomic sequence targeted by the gRNA showing example of mutations. ‘‘-’’ indicates nucleotide deletions. wt: wild-type sequence. *Numbers in parentheses* indicate the number of clones detected during sequencing.
